# Artificial intelligence in head and neck cancer: a bibliometric analysis of research landscape, emerging trends, and challenges

**DOI:** 10.3389/fonc.2025.1604136

**Published:** 2025-09-01

**Authors:** Shufang Liu, Jingdan Zhang, Ziye Tan, Bo Zhou

**Affiliations:** ^1^ Hunan Cancer Hospital / The Affiliated Cancer Hospital of Xiangya School of Medicine, Central South University, Changsha, China; ^2^ Xiangya School of Medicine, Central South University, Changsha, Hunan, China

**Keywords:** head and neck cancer, artificial intelligence, deep learning, bibliometric, tumor

## Abstract

**Background:**

Head and neck cancer is the seventh most common cancer worldwide. As an aggressive malignancy, it is characterized by high metastasis rates, complex anatomy, challenging treatments, high recurrence rates, and significant disability. Over the past decade, advancements in big data, AI algorithms, and hardware have enabled artificial intelligence to make substantial contributions to addressing medical challenges in oncology, including head and neck cancer. The era of AI-driven head and neck tumor management may soon arrive. Despite significant attention, there has been a lack of quantitative literature-based studies in this field.

**Objective:**

This study aims to delineate the knowledge structure, hotspots, and trends in AI applications for head and neck cancers since 1995 through bibliometric analysis.

**Method:**

We conducted a comprehensive literature search via the Web of Science, utilizing tools such as CiteSpace, ArcGIS, and VOSviewer for analysis, with a focus on key countries, institutions, authors, and emerging topics.

**Result:**

We analyzed 362 papers authored by 235 researchers from 189 institutions across 55 countries, with China leading in publication output. Radiotherapy and Oncology was the most influential journal. Bur, Andres M was the pioneering author, and the University of Texas System ranked as the top publishing institution. Currently, the most significant keywords include “target volumes,” “prognosis,” “algorithm,” “survival,” “lesions,” and “automatic diagnosis.” Additionally, we identified 12 keyword clusters in the field, with the latest five clusters labeled as “automatic diagnosis”, “explainable artificial intelligence”, “guidelines”, “research trends”, and “natural intelligence”.

**Conclusion:**

This article provides a concise overview of the current landscape and emerging trends in AI applications for head and neck cancer research, offering insights and guiding future studies in this evolving field.

## 1 Introduction

Head and neck cancers (HNCs) represent a heterogeneous group of malignancies originating from the mucosal surfaces of the upper aerodigestive tract. According to the latest Global Cancer Statistics (GLOBOCAN), HNCs rank as the seventh most prevalent malignancy worldwide, comprising approximately 3% of all new cancer diagnoses and 1.5% of cancer-related mortality (1). These aggressive neoplasms are characterized by high metastatic potential, frequent recurrence rates,and high disability rate. Due to the complexity of the maxillofacial anatomical structure, difficulties associated with surgical access, and the challenging protection of structural functions, tumors are often difficult to treat and eradicate through surgery, frequently requiring adjuvant standard radiotherapy or chemotherapy. These therapeutic complexities profoundly impact patients’ quality of life and impose substantial socioeconomic burdens. Early detection and accurate staging are pivotal for improving clinical outcomes in HNC management (2). However, the majority of patients present with advanced-stage disease due to anatomical obscurity of primary lesions and nonspecific early symptomatology. Conventional diagnostic paradigms relying on imaging modalities (CT, MRI, PET-CT), endoscopic biopsy, and molecular marker analysis demonstrate suboptimal sensitivity and diagnostic accuracy, particularly for early-stage lesions (2). These limitations underscore the critical need for innovative diagnostic strategies to enable timely intervention and personalized treatment approaches. Consequently, there is an urgent requirement within the clinical community for more precise diagnostic technologies and targeted therapeutic modalities to address these unmet medical needs.

In 1956, the term “Artificial Intelligence” (AI) was first coined by McCarthy at the Dartmouth Conference (3). This technology refers to programmed systems capable of identifying patterns and establishing input-output correlations, enabling evidence-based decision making for novel data inputs (3). Core AI architectures include Machine Learning (ML), Deep Learning (DL) with Convolutional Neural Networks (CNN), and Natural Language Processing (NLP) (4). Deep Learning (DL) is a subdomain of artificial intelligence, characterized by its ability to perform automatic feature extraction and exhibit robust capabilities in learning from and evaluating large amounts of complex data. The recent decade has witnessed remarkable progress in big data analytics, optimized AI algorithms, and networked computing, collectively empowering AI applications in oncology management particularly for head and neck cancers (5). CNN-powered DL architectures demonstrate clinical value in automated feature extraction from medical imaging, enhancing pathological classification for cancer diagnosis and grading (6). Notably, Deep learning (DL) algorithms can characterize potential genetic and epigenetic heterogeneity using histopathological images. When tumor tissue samples are not available for mutation analysis, features in histopathological images can predict genetic mutation points and provide biomarker information for immunotherapy, which is more accessible and economical than direct sequencing (5). The artificial intelligence treatment decision model can predict the treatment response and survival rate of patients with advanced head and neck cancer to chemotherapy. Additionally, it can formulate personalized treatment plans based on patients’ conditions and guidelines, thereby achieving precision medicine (7). Radiotherapy optimization represents another pivotal application, where AI automates tumor target contouring and dynamically adjusts radiation dosage, enhancing both therapeutic precision and clinical workflow efficiency (8). The emergence of explainable AI (X-AI) provides clinician-interpretable decision pathways, allowing expert validation of algorithmic outputs and systematic error correction - critical safeguards for clinical practice. Current evidence confirms AI’s expanding role across diagnostic classification, phenotypic characterization, therapeutic response prediction, and prognostic modeling in head and neck oncology. The era of AI-driven head and neck cancer management may be in the near future.

The application of artificial intelligence in head and neck cancer represents a complex and profound research domain. Given the diverse pathological types, anatomical complexities, and high disease heterogeneity of head and neck cancers, numerous scholars have generated substantial research outcomes through various AI methodologies and from different perspectives and levels (9). However, this thriving field still lacks a comprehensive summary from a macro perspective. With the emergence of numerous AI tools, it is timely and insightful to conduct a macro-level academic visualization and quantitative analysis of AI applications in head and neck cancer. This exploration aims to discuss the cutting-edge advancements and future applications of AI in the field of head and neck cancer.

This study aims to conduct a comprehensive and visual bibliometric analysis of the application of artificial intelligence in head and neck cancer. It elucidates the research landscape and domain structure related to artificial intelligence and head and neck cancer globally, including authoritative countries, prolific institutions, and high-contributing authors. The study summarizes keyword clusters, trends, and cutting-edge topics, exploring popular research subjects and their unique attributes in this field. By promoting academic exchanges between clinicians and researchers, it aims to facilitate further exploration, ultimately providing more and better diagnostic and therapeutic assistance to patients with head and neck cancer. Additionally, based on the analysis of emerging trends and research structures, this study examines potentially valuable research directions, offering new ideas and insights for the development of artificial intelligence in the field of head and neck cancer.

## 2 Method

### 2.1 Research methods

Bibliometrics, as a quantitative analytical approach, employs multiple extrinsic attributes of scientific literature as analytical targets (10). It applies mathematical and statistical methodologies to evaluate, describe, and forecast the current status and future trajectories of scientific and technological development (10). These bibliometric techniques are instrumental in uncovering latent knowledge frameworks embedded within scholarly publications, such as keyword distributions and citation patterns (11). Furthermore, they facilitate the synthesis and graphical representation of research outputs, thereby fostering a profounder comprehension of specific academic domains (12).

CiteSpace is an information visualization tool based on citation analysis theory developed by Dr. Chen Chaomei from Drexel University. It can explain the structure, patterns, and distribution of scientific knowledge, producing the so called “scientific knowledge graph” (13). CiteSpace is mainly used to organize theoretical viewpoints, track evolutionary paths, predict development trends, conduct in-depth research on academic history, and identify current research hotspots within a particular field (13). It is a practical quantitative analysis tool used to scrutinize research documents (11).

CiteSpace analysis methods include co-citation analysis, co-occurrence analysis, burst detection, and cluster analysis (13). Co-citation analysis consists of examining the relationship between two studies based on their co-citation in the third study. Studies that are frequently cited together are considered to be more similar and interrelated. Co-occurrence analysis the number of times that a particular keyword appears in the literature of a given field, and measures the correlation between them by co-occurrence. Burst detection can identify fluctuations in the use of particular keywords, while cluster analysis groups objects on the basis of their similarities, helping to analyze multiple clusters formed (14–17).

Centrality is a critical metric that gauges the importance of an object in a network. Nodes with an intermediate centrality value greater than 0.1 are called central nodes or key nodes, which have an important influence in the research field and are often act as bridges connecting different kinds of research objects like articles, keywords, and countries (18).

CiteSpace is uniquely positioned to pinpoint pivotal aspects and future directions in the field of research, making it an invaluable tool for scholarly investigations (13). In this study, the bibliometrics analysis software CiteSpace was deployed to analyze the existing literature on AI in orthopedics. Critical readings were also conducted to delve into key research findings and provide vital insights into this subject matter.

This study utilized CiteSpace and VOSviewer for visualizations (13, 19), Detailed descriptions of the software principles and academic terminology definitions can be found in the **Supplementary File 1**.

### 2.2 Data resource

In this study, the Web of Science Core Collection (WoSCC) served as the data source, with the indexing source being the Science Citation Index Expanded (SCIE). The search strategy employed here is presented below: (TS=(“artifici* intelligen*” OR “open* ai” OR “intelligen* artifici*” OR “chatgpt*”)) AND TS =((“head*” OR “neck*” OR “oral*” OR “parasonal* sinus*” OR “nasal* cavit*” OR “sinonasal*” OR “nasopharyn*” OR “oropharyn*” OR “hypopharyn*” OR “laryn*” OR “salivary*”) AND (“cancer*” OR “carcinoma*” OR “oncolog*” OR “tumor*”)). All electronic searches were performed on January 1^st^, 2025 in China. A total of 842 articles were searched, 548 original articles were selected and written in English. In this phase, a triple-blind evaluation process was implemented for the originally retrieved literature, adhering to predefined inclusion and exclusion criteria. To avoid being influenced by author/institution preferences, journal/source biases, and interference from prior cognition, and to ensure that literature screening is based solely on objective criteria such as relevance to the research topic and data integrity, we conducted a triple-blind literature screening. The specific process is as follows: Before the evaluation, non-content information of the literature—including author names, affiliations, journal sources, publication dates, and citation data—was anonymized, with only abstracts, keywords, and core fragments of the research content retained. This ensures that evaluators focus entirely on the match between the literature and the research topic. Three researchers from relevant fields (with no conflicts of interest) conducted independent screening separately, unaware of each other’s evaluation results, to avoid consensus bias caused by “group thinking”. To ensure relevance, only literature that received unanimous approval—i.e., all experts agreed it is relevant to the research field—was included in the literature pool. The three reviewers are oncologic PhDs from three different provinces in China.

**Figure 1:**
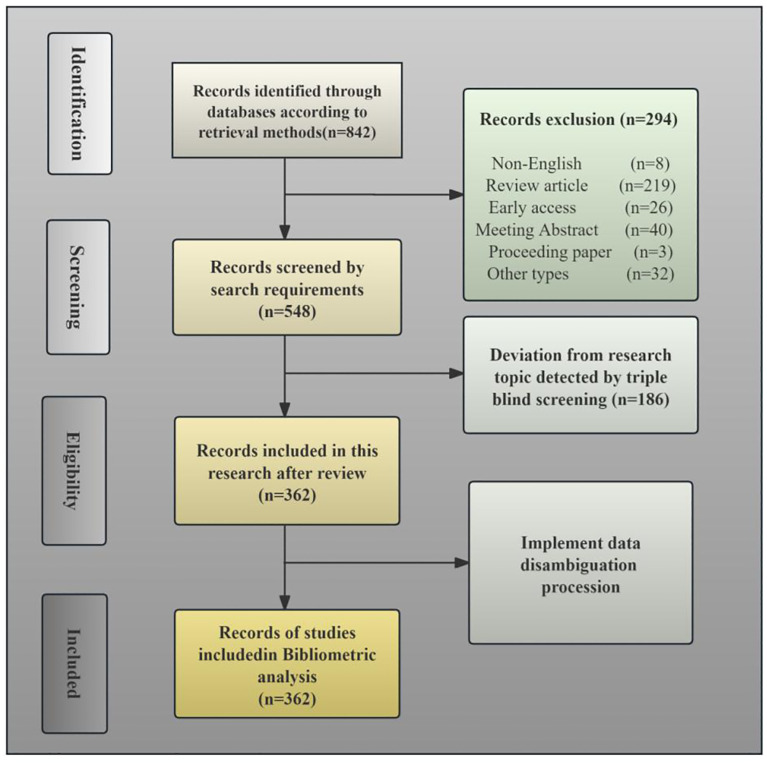
PRISMA flow diagram illustrating the search strategy employed in the current bibliometric study.

Ultimately, 362 non-redundant articles were included for bibliometric examination. **Figure 1** presents the PRISMA flow diagram outlining the systematic literature search and screening methodology.

#### 2.2.1 Inclusion criteria

1. The search is limited to publications which are written in English and have complete reference and citation records.
2. Studies must explicitly investigate head and neck malignancies, with scientific focus areas including but not limited to: diagnostic imaging interpretation, therapeutic protocol development, anatomical mapping, prognostic modeling, or treatment efficacy assessment specific to head and neck cancer.
3. The research content is closely associated with Artificial Intelligence or its critical components, including neural networks, deep learning, natural language processing, and large-scale database mining.

#### 2.2.2 Exclusion criteria

1. Non-English publications or records with incomplete bibliometric metadata (e.g., missing author affiliations, citation records, or indexing information).
2. Preclinical studies utilizing non-human biological models (e.g., veterinary/zoological research involving canine, bovine, feline, or porcine subjects, etc.).
3. Articles containing only incidental mentions of Artificial Intelligence (AI), such as statements like “This article’s abstract/main text/images were generated by AI and manually reviewed/edited” or “We believe this finding could be combined with AI in the future,” but where the experimental design, research methods, or subjects are unrelated to AI or its core components.
4. Similarly, articles containing coincidental mentions of head and neck cancer (HNC)-related keywords (e.g., “supraclavicular metastasis in gastric cancer,” “cervical lymph node metastasis in breast cancer”) but where the actual research content does not involve primary HNC will be excluded. Example: “Breast cancer demonstrates high cervical lymph node metastasis rates … The objective of our study is AI-assisted breast cancer imaging screening.”

## 3 Results

### 3.1 Analysis of publishing trend

**Figure 2:**
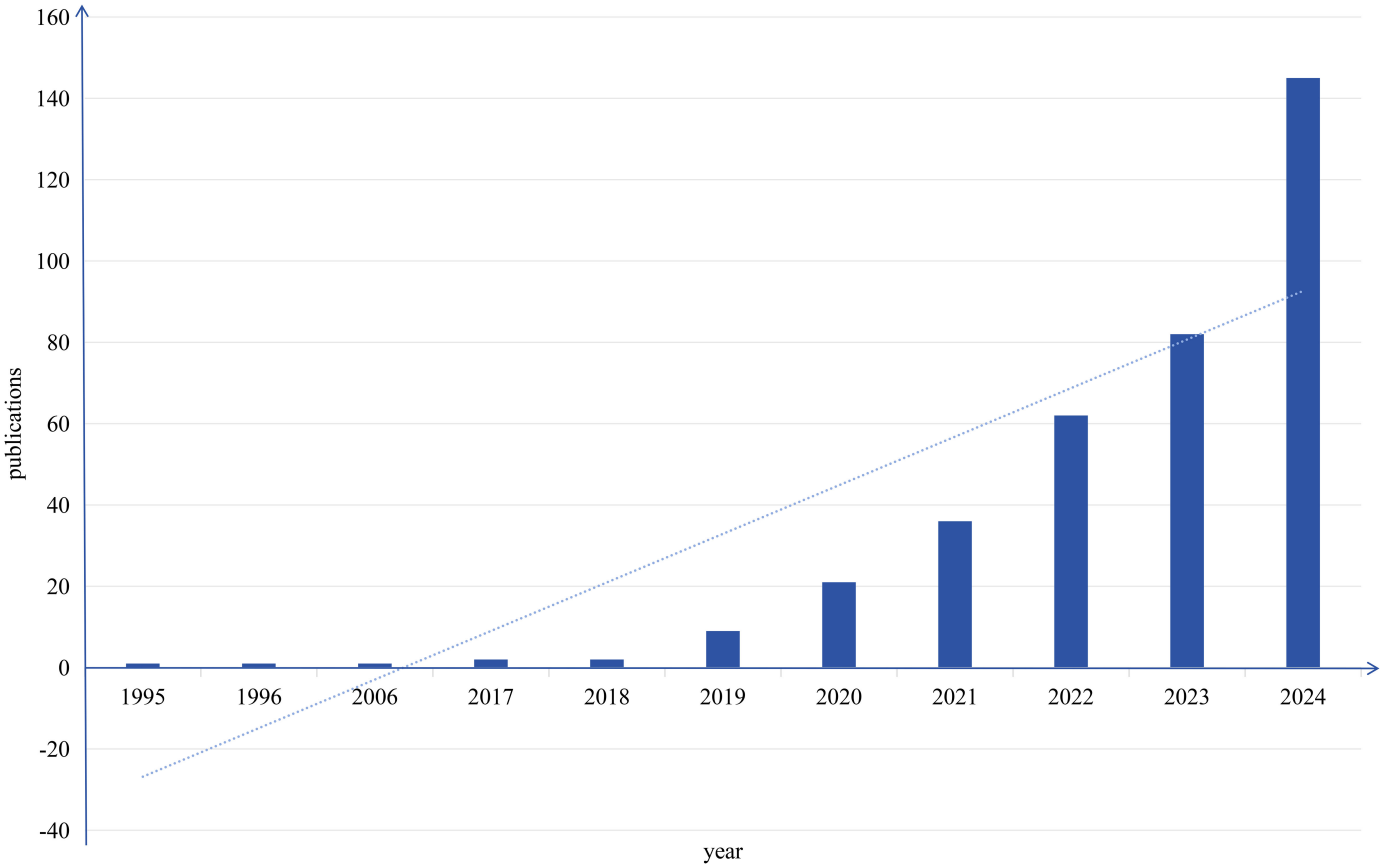
Time evolution of the total number of publications in the WOS database.

The pattern of scholarly output serves as a critical metric for assessing disciplinary advancement in specific research areas. Therefore, constructing a publication output timeline enables systematic assessment of current research landscapes and prediction of future trajectories. **Figure 2** depicts the yearly publication counts of studies intersecting artificial intelligence and head and neck cancer obtained via Web of Science. This analysis revealed 362 eligible studies published since 1995, with mean annual productivity of 12 papers. The temporal distribution demonstrates two clear evolutionary stages, with 2019 acting as the key inflection point.

During the first phase (1995–2018), only 7 papers were published, averaging 0.29 publications per year. In contrast, the second phase (2019–2023) witnessed exponential growth, with 355 publications produced at an annual rate of 59.16 papers. This remarkable growth trajectory aligns with key milestones in medical AI development. While early-stage applications remained largely exploratory, recent advances in computational hardware and algorithmic optimization have accelerated AI’s integration into head and neck cancer research, particularly in precision medicine approaches. Current publication trends suggest sustained expansion of this field in the foreseeable future.

### 3.2 National, institutional, author and journal analysis

#### 3.2.1 National analysis

**Figure 3:**
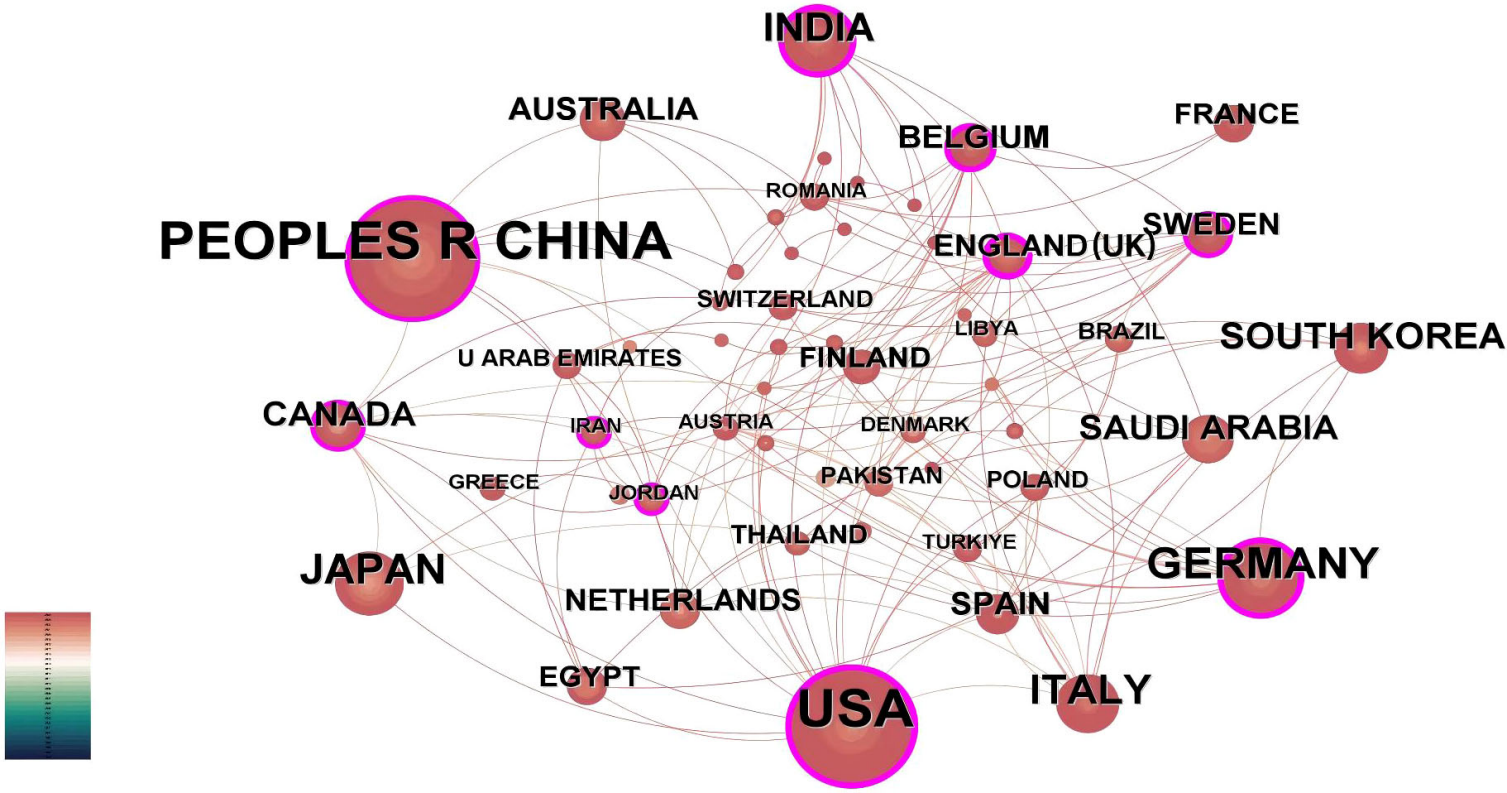
Country-level publication analysis. International collaboration network. The network spans 1995–2024 (1-year intervals). Nodes denote countries, scaled by publication counts. Purple outlines highlight nations with intermediary centrality >0.1, reflecting their connective role in cross-border collaborations.

Country-level quantitative assessment not only identifies key nations in AI-integrated head and neck cancer research but also reflects scholarly exchanges and collaborative dynamics among these regions. Using CiteSpace’s “country” parameter, this study generated a collaboration network consisting of 55 nodes, 153 links, and a density score of 0.103 (**Figure 3**). Analysis of publication patterns highlights distinct national contributions in AI-driven head and neck oncology. **Table 1** shows that China leads in publication output with 87 articles, closely trailed by the United States (84), Germany (34), Japan (30), and Italy (28). However, international collaboration metrics reveal a different landscape: The United States demonstrates the highest research interconnectedness (0.38), with England (0.35) and India (0.29) emerging as additional hubs of multinational cooperation. They have established robust international collaboration networks, correlating with their disproportionately high output of influential studies. In contrast, countries including Japan, South Korea, France, and Thailand exhibit near-zero centrality scores (approaching 0), reflecting predominantly independent research paradigms. Historical analysis further identifies the United States and England as pioneering nations in this field, their earliest investigative efforts laying essential groundwork for subsequent global advancements in AI applications for head and neck oncology.

**Table 1:** The frequency and centrality of publication in countries/regions.

Country	Year of first publication	Frequency	Centrality
PEOPLES R CHINA	2019	87	0.18
USA	1996	84	0.38
GERMANY	2019	34	0.21
JAPAN	2019	30	0.01
ITALY	2017	28	0.06
INDIA	2020	23	0.29
SOUTH KOREA	2020	18	0.02
CANADA	2018	15	0.11
SAUDI ARABIA	2021	15	0.05
SPAIN	2019	12	0.08
BELGIUM	2019	11	0.2
AUSTRALIA	2021	11	0.09
UK	2006	10	0.35
SWEDEN	2020	10	0.14
NETHERLANDS	2020	10	0.07
FRANCE	2022	9	0
FINLAND	2020	9	0.04
EGYPT	2022	9	0.05
THAILAND	2020	7	0.01
PAKISTAN	2022	5	0.08

#### 3.2.2 Institutional analysis

**Figure 4:**
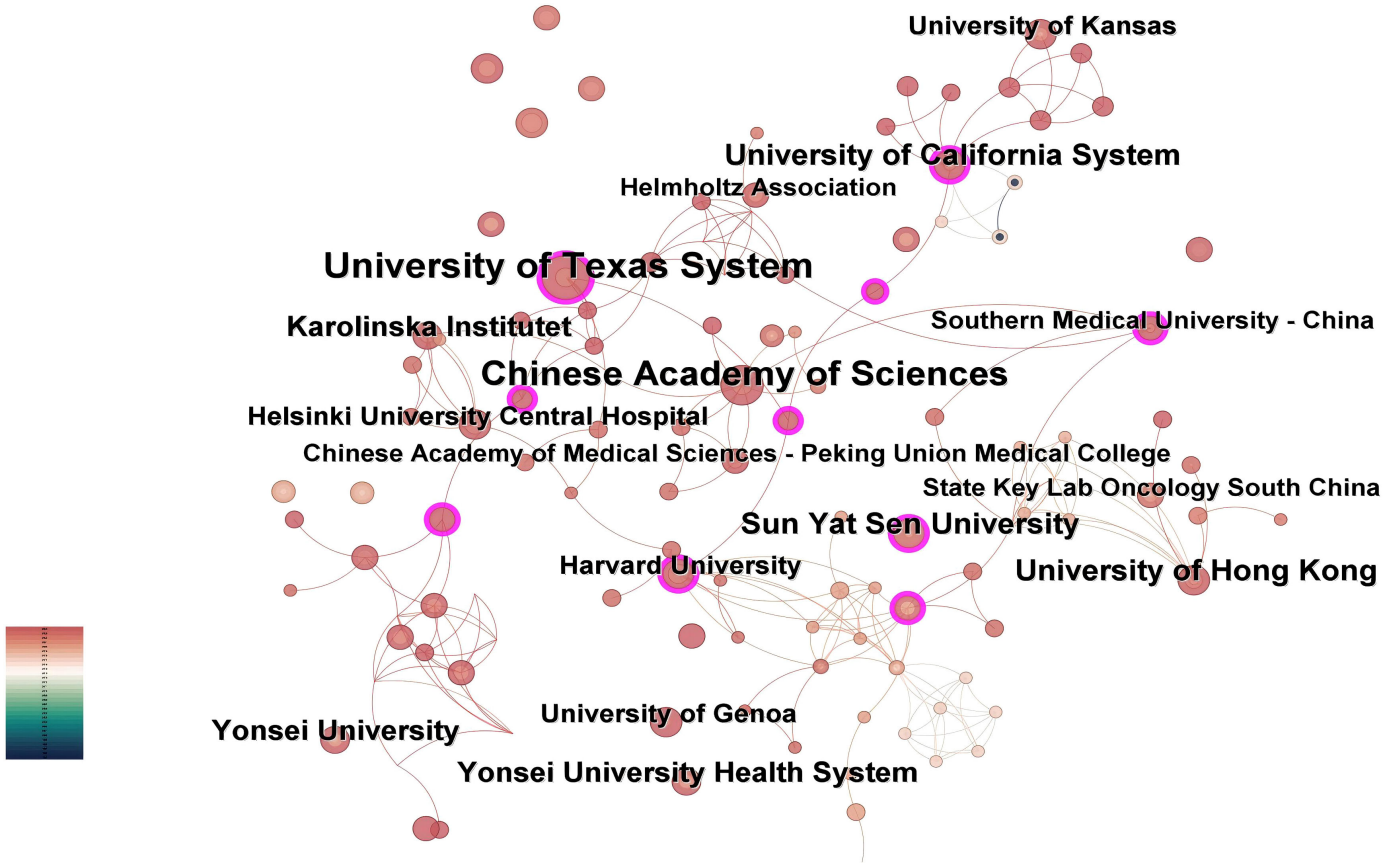
Institutional co-occurrence map. Nodes represent academic institutions, with size proportional to publication output. Purple-ringed nodes denote entities exhibiting high brokerage potential (betweenness centrality >0.1) within the cooperative landscape.

The institutional collaboration network generated by CiteSpace analysis (**Figure 4**) comprises 189 nodes and 308 connections, with a network density of 0.0173. Institutional analysis reveals concentrated research productivity in Guangdong Province, China and the Southern United States - regions characterized by elevated head and neck cancer incidence rates (20). As evidenced in **Table 2**, the University of Texas System leads with 15 publications, followed by the University of Hong Kong (8 publications), Sun Yat-sen University (8 publications), and the University of California System (8 publications). Three institutions demonstrate critical bridging roles: University of Texas System (centrality=0.16), Sun Yat-sen University (centrality=0.17), and University of California System (centrality=0.12). Conversely, 98.4% of institutions exhibit centrality values below 0.1. The higher the centrality, the more frequently the institution lies on the cooperation paths between other institutions, serving as a key node for promoting cross-group knowledge flow and resource integration. In contrast, 98.4% of institutions in this study have a centrality of < 0.1, which means that these institutions are mostly confined to closed small-scale cooperation networks (such as collaboration within the same region or the same institution) and rarely play the role of a ‘bridge’ connecting different cooperation groups. Due to the lack of cross-group connections, local innovative achievements are difficult to spread to a wider research network, potentially missing opportunities for breakthroughs brought by interdisciplinary integration (such as the integration of medicine and engineering in the development of AI algorithms);The technical advantages of different institutions (such as clinical data accumulation and algorithm research and development capabilities) cannot be complemented through cooperation, which may lead to repetitive research or low innovation efficiency.

**Table 2:** The top 20 institutions of publication.

Institution	Year of first publication	Frequency	Centrality
University of Texas System	2021	15	0.16
University of Hong Kong	2019	8	0.04
Sun Yat Sen University	2019	8	0.17
University of California System	2017	8	0.12
Yonsei University Health System	2020	7	0.02
Karolinska Institutet	2020	7	0.01
Yonsei University	2020	7	0.02
Egyptian Knowledge Bank (EKB)	2022	7	0
Chinese Academy of Sciences	2021	7	0.05
Harvard University	2020	6	0.06
University of Kansas Medical Center	2019	6	0
University of Genoa	2022	6	0.01
University of Kansas	2019	6	0
King Khalid University	2022	6	0
Chinese Academy of Medical Sciences - Peking Union Medical College	2022	6	0.01
Helsinki University Central Hospital	2020	6	0.03
University of Texas Southwestern Medical Center Dallas	2023	6	0
Karolinska University Hospital	2020	6	0.01
University of Tokyo	2020	5	0
Harvard Medical School	2020	5	0.12

Notably, the University of Texas System achieved its leading position through accelerated productivity between 2021-2025, despite later entry into the field. This rapid ascension correlates with its establishment of strategic international partnerships, as reflected in dense collaboration linkages within the network.

#### 3.2.3 Author analysis

**Figure 5:**
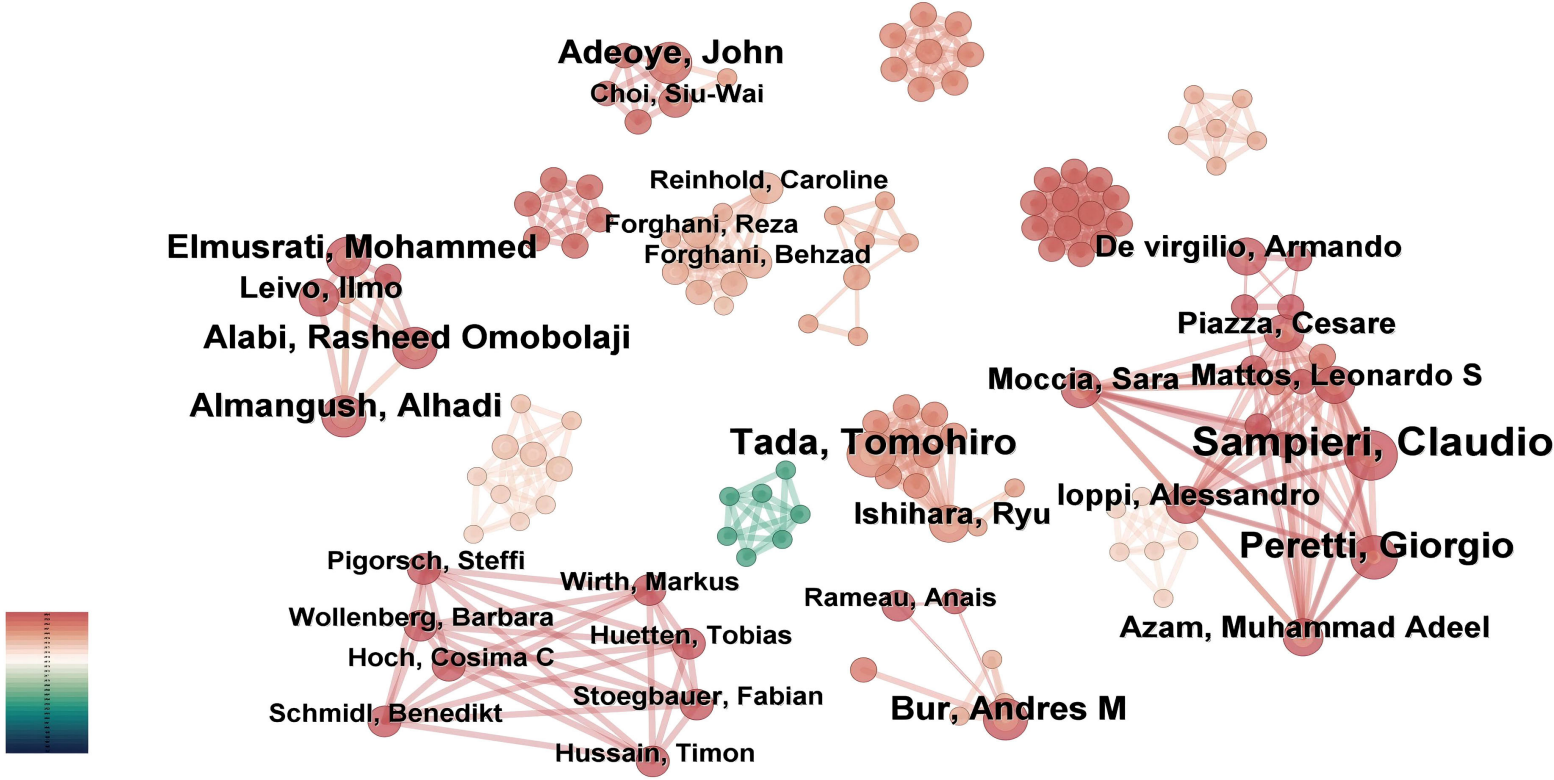
Author-centric network mapping. CiteSpace constructed co-authorship relationships, where node size is scaled by individual publication output.

Based on the “authors” parameter analysis in CiteSpace, **Figure 5** illustrates an author collaboration network consisting of 235 nodes, 527 links, and a density score of 0.0192. **Table 3** enumerates the top ten researchers ranked by their publication outputs.

**Table 3:** Top 10 authors in publication count.

Author	Year of first publication	Frequency
Sampieri, Claudio	2022	8
Tada, Tomohiro	2020	6
Peretti, Giorgio	2022	6
Almangush, Alhadi	2020	5
Adeoye, John	2020	5
Alabi, Rasheed Omobolaji	2020	5
Elmusrati, Mohammed	2020	5
Bur, Andres M	2019	5
Piazza, Cesare	2022	4
Moccia, Sara	2022	4

The three most prominent nodes in the visualization map correspond to Sampieri, Claudio (8 publications), Tada, Tomohiro (6 publications), and Peretti, Giorgio (6 publications), signifying their significant contributions to this interdisciplinary domain. Among these prolific scholars, Bur, Andres M from the University of Kansas Medical Center (USA), a board-certified otolaryngology-head and neck surgeon, emerged as the trailblazer who pioneered the application of machine learning algorithms for predicting lymph node metastasis and disease prognostication in head and neck cancer. His foundational research established a robust theoretical framework for AI translational medicine and provided critical impetus for subsequent innovations.

#### 3.2.4 Journal co-citation analysis

**Figure 6:**
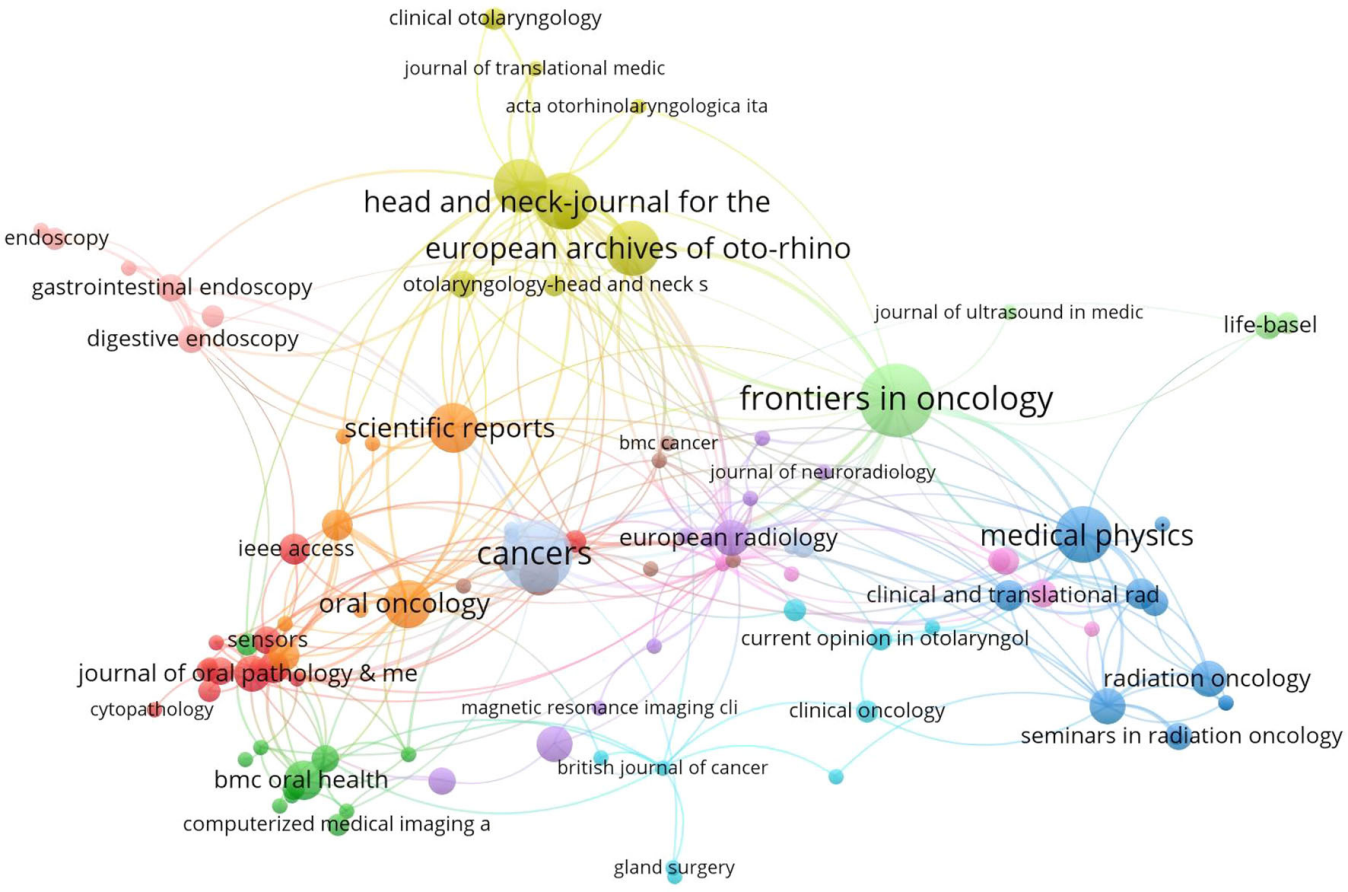
Analysis diagram of journal co-citation network.

Through bibliometric analysis of scientific citations using VOSviewer, we systematically identified the most influential journals at the intersection of artificial intelligence and head and neck oncology. As illustrated in **Figure 6**, the node sizes (representing publication volume) reveal *Frontiers in Oncology*, *Cancers*, and *Medical Physics* as the three predominant publication platforms in this domain. This suggests that manuscripts focusing on AI applications in head and neck cancer management demonstrate higher acceptance potential in these periodicals. Complementing these findings, **Table 4** enumerates the most frequently referenced journals, where *Radiotherapy and Oncology* (307 citations), *Cancers* (261 citations), and *Gastrointestinal Endoscopy* (241 citations) emerge as the most impactful sources. Although the number of articles in these journals is limited, they hold relatively more influence and significance in shaping the development of the field. In 2024, *Frontiers in Oncology* and *Cancers* journals published over 20 articles in the field of artificial intelligence and head and neck cancer, indicating a noticeable increase in attention to this area.

**Table 4:** Journals with the Top10 citation numbers.

Rank	Journals	Publications	Citations	Average citation counts
1	Radiotherapy And Oncology	5	307	61
2	Cancers	20	261	13
3	Gastrointestinal Endoscopy	3	241	80
4	Laryngoscope	11	232	21
5	Physics In Medicine and Biology	4	232	58
6	Radiology	1	227	227
7	Frontiers In Oncology	21	216	10
8	Oral Oncology	9	203	23
9	Medical Physics	13	197	15
10	European Radiology	5	160	32

### 3.3 Keywords cluster, keywords burst and frontiers analysis

#### 3.3.1 Keywords cluster

**Figure 7:**
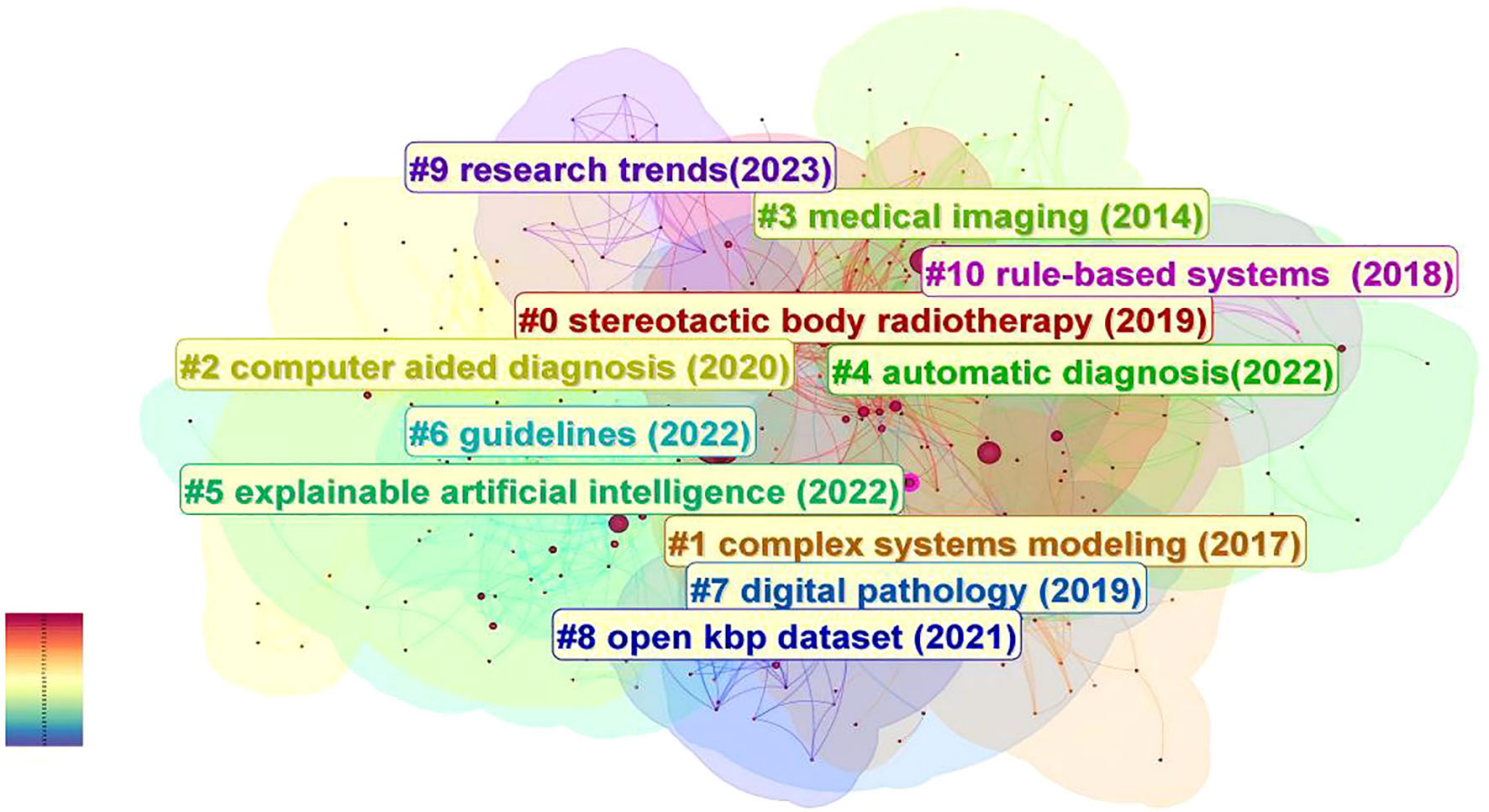
The analysis of keywords clustering, which was conducted by selecting the “cluster” option and employing the Pathfinder algorithm to draw connection lines, ensuring the rationality of cluster classification. A total of 12 keywords clusters were identified, with each cluster assigned a distinct color based on the timestamp in the bottom left corner. The names of the clusters are derived from a set of representative keywords obtained using the LLR algorithm.

In this study, we utilized Citespace to group keywords by selecting the “cluster”. The results are illustrated in **Figure 7** in details, which depicts the research topics related to the field with a total of 12 clusters.

These clusters are as follows: #0 stereotactic body radiotherapy (2019), #1 complex systems modeling (2017), #2 computer aided diagnosis (2020), #3 medical imaging (2014), #4 automatic diagnosis (2022), #5 explainable artificial intelligence (2022), #6 guidelines (2022), #7 digital pathology (2019), #8 open kbp dataset (2021), #9 research trends (2023), #10 rule-based systems (2018), #11 natural intelligence (2022).

Chronological analysis of keyword clusters revealed Cluster #3 as the earliest emerging group, with an average keyword occurrence year of 2014. During this formative period, artificial intelligence (AI) transitioned into clinical implementation, witnessing growing recognition of deep learning techniques in medical image recognition and processing. Researchers commenced foundational investigations into convolutional neural networks (CNNs) for medical image classification, concurrent with early developments in large-scale medical imaging databases. Emerging clinical evidence began validating AI’s potential across prevention, detection, diagnostic accuracy, and therapeutic interventions in head and neck oncology. Beside Cluster #3, Early-stage clusters included Cluster #1 (2017) and Cluster #10 (2018), characterized by the representative term “complex systems modeling” – referring to systems comprising multiple interacting components that exhibit nonlinear dynamics. This methodological approach enabled simulation of head and neck cancer epidemiology, including risk factor interactions and disease transmission patterns. The modeling framework further permitted analysis of cellular interactions within tumor microenvironments, informing personalized treatment selection and outcome prediction.

Clusters #0 and #7 (2019 average year) demonstrated expanding clinical applications through representative terms like “stereotactic body radiotherapy” and “digital pathology”. AI implementations enabled automated tumor segmentation with real-time spatial localization capabilities, enhancing radiotherapy precision through optimized beam positioning and dosage calculation while minimizing healthy tissue exposure. Concurrent applications emerged in lymph node metastasis detection, immunohistochemical analysis, biomarker identification, and histopathological image interpretation, establishing critical roles in digital pathology workflows. Cluster #2 and Cluster #4 mainly involve the screening, diagnosis, and differential diagnosis of head and neck cancer. Around 2020, remarkable progress was achieved in computer-aided diagnosis, with continuous expansion of clinical applications and a significant improvement in real-time analysis capabilities. By around 2022, the development of fully automated diagnostic systems, the construction of integrated platforms, the continuous optimization of self-learning abilities, and the large-scale application of big data technologies, etc., all promoted the further development of AI in the field of automatic diagnosis of head and neck cancer. Moreover, around 2022, the research focus of scientists also included the differential diagnosis of head and neck cancer, such as potentially malignant oral lesions and premalignant lesions like oral leukoplakia, oral lichen planus, and laryngeal nodules. The average year of Cluster #8 is around 2021, and the representative keyword is “open kbp dataset”. During this period, a large number of medical datasets were expanded and optimized. The combination of artificial intelligence (AI) with large datasets (such as the open-source KBP dataset, Radiomics dataset, etc.) has promoted the optimization of imaging analysis, diagnosis, and treatment plans, as well as the development of clinical decision-making and prediction support systems. The years of Cluster #5, #6, and #11 are 2022. Explainable artificial intelligence is an AI model that can provide a clear and transparent decision-making process, enabling experts and clinicians to understand and trust the decisions made by AI, and helping to identify potential biases and errors in the model to ensure the safety and effectiveness of medical decisions. Cluster #6, with “Guidelines” as the representative keyword, reminds us that during the development of artificial intelligence related to head and neck cancer, supervision and standardization are important aspects to ensure its safety and effectiveness. Medical regulatory agencies in various countries are gradually realizing the importance of formulating relevant standards and guidelines. The compatibility and interoperability among different artificial intelligence systems, data sharing standards, quantification of performance indicators, relevant regulatory laws and regulations, and ethics, etc., will become one of the key focuses in the development of artificial intelligence in the field of head and neck cancer.

Regarding Cluster #11, the representative keyword is “natural intelligence”, which indicates that in recent years, scientists often use natural intelligence as a reference for comparison and a source of inspiration. They draw on the principles and mechanisms of natural intelligence to develop new artificial intelligence algorithms and models.

The latest Cluster #9, “research trends” (2023), includes keywords such as “drug-resistant cancer” and “texture analysis”, which herald that the future applications of artificial intelligence in the field of head and neck cancer will become more complex and refined. 

#### 3.3.2 Hotspots analysis

**Figure 8:**
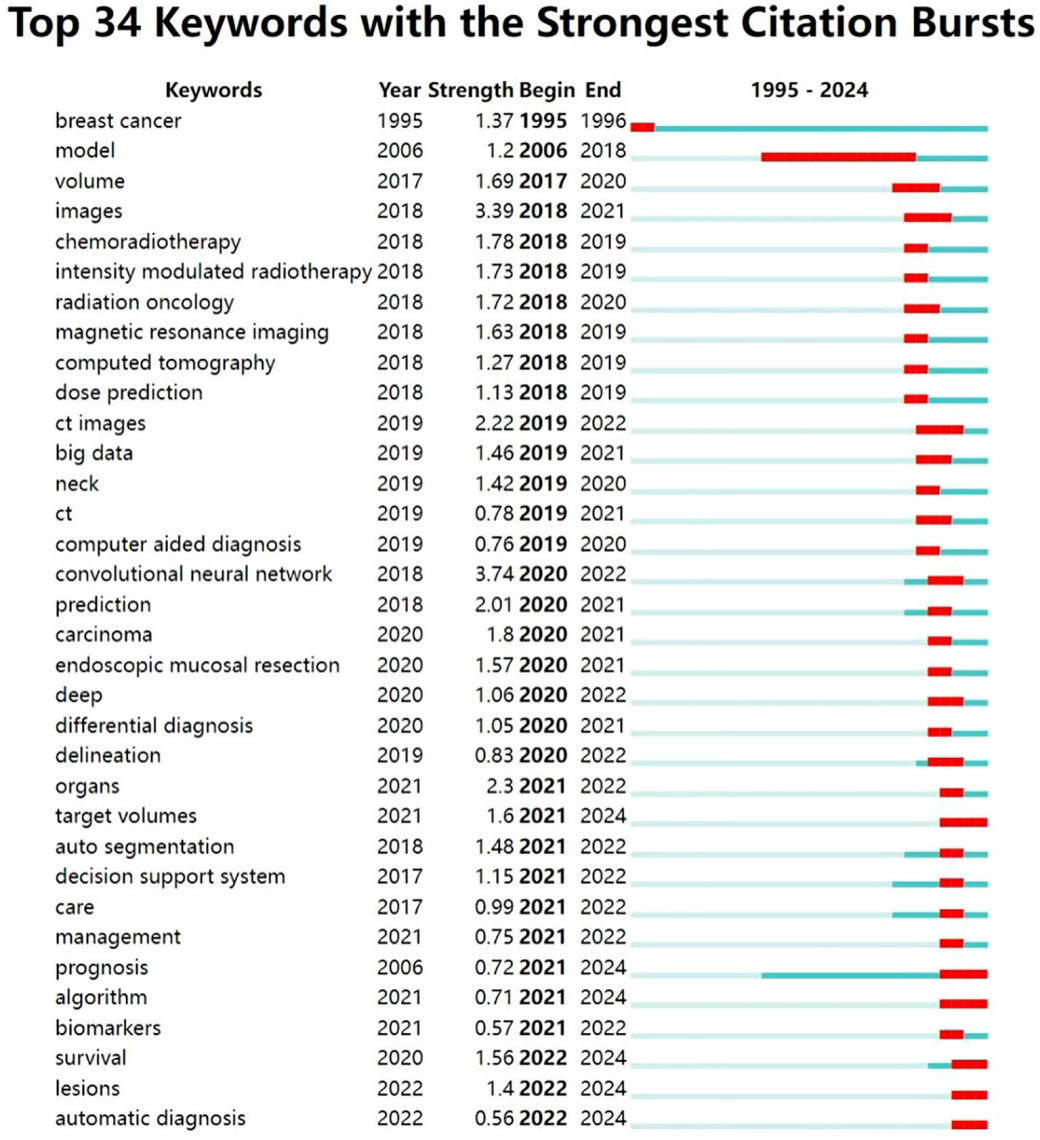
Top 30 keywords with the strongest citation bursts.

In this study, we used the burst detection algorithm in CiteSpace to analyze the evolution of research hotspots related to the application of artificial intelligence (AI) in head and neck cancer based on data retrieved from the Web of Science Core Collection. The resulting visualization (**Figure 8**) highlights the top 30 keywords with the strongest citation bursts, showing both their intensity and duration. In the figure, the blue lines represent the full timeline, while the red segments indicate periods of concentrated attention for each keyword. Recently, keywords such as “target volumes,” “prognosis,” “algorithm,” “survival,” “lesions,” and “automatic diagnosis” have shown particularly strong bursts, reflecting current research priorities.

Based on the temporal distribution of these keywords, we divided the field’s development into two main stages, using 2020 as a turning point.

In the initial stage, foundational technologies and research frameworks began to emerge. Keywords such as “model” and “computed tomography” suggest a focus on theoretical groundwork and the integration of AI with radiological imaging. Imaging technologies—including “magnetic resonance imaging,” “images,” and “CT images”—were among the earliest areas to draw attention in the context of AI applications. These tools laid the foundation for AI-supported image analysis, which in turn enabled new possibilities in clinical care.

Radiotherapy also began to benefit from AI during this phase. Owing to its capacity to analyze imaging data, AI helped distinguish between pathological and healthy tissues and enabled more accurate dose estimation. These improvements helped reduce damage to surrounding healthy structures and improved the overall efficiency of treatment. Keywords such as “chemoradiotherapy,” “intensity-modulated radiotherapy,” and “dose prediction” reflect this growing interest.

Toward the end of this phase, the emergence of the keyword “big data” signaled rapid progress in data mining techniques. The expansion and standardization of large datasets provided critical support for AI development by offering the necessary volume and quality of training data. At the same time, studies related to “computer-aided diagnosis” began to increase significantly. These AI-driven systems offered real-time decision support and data integration, helping clinicians identify lesions more quickly, improve early detection, and streamline diagnostic processes.

After 2020, the application of AI in head and neck cancer entered a period of rapid expansion. Theoretical frameworks and algorithmic methods became more refined, as reflected in sustained bursts of keywords such as “convolutional neural network,” “deep,” and “algorithm.” These terms suggest ongoing interest in the underlying AI architecture and signal their continued relevance throughout the field’s development.

In diagnostic contexts, AI applications have become more sophisticated and automated. Keywords like “differential diagnosis” and “automatic diagnosis” indicate a shift toward greater accuracy and reliability, especially in distinguishing between diseases with similar presentations. AI systems increasingly support full automation in medical data integration and analysis, thereby reducing clinicians’ workload and enhancing diagnostic workflows.

At the same time, greater attention has been paid to predicting outcomes and evaluating treatment responses. Keywords such as “prediction,” “prognosis,” and “survival” show a clear trend toward using AI to support prognostic modeling and personalized treatment planning. These developments have helped lay the groundwork for “decision support systems” that improve clinical information integration and decision-making in practice.

The emergence of “endoscopic mucosal resection” and “biomarker” as burst terms suggests further progress toward precision and personalized medicine. AI technologies are now being used to assist with complex clinical procedures and to guide individualized therapeutic strategies.

Finally, the recent appearance of the keyword “management” underscores the growing importance of system-level governance in AI applications. In the context of head and neck cancer, AI management involves not only patient care, data integration, clinical trial oversight, and workforce training, but also essential concerns such as ethical standards, data privacy, and technical interoperability. Effective oversight ensures that AI technologies are applied safely and responsibly, supporting long-term progress in both clinical practice and healthcare systems as a whole.

## 4 Discussion

In this study, we conducted a literature search in WoSCC and included 362 published articles matching the theme. This paper summarizes the development trends and hotspots of AI in the field of head and neck cancer, examining potentially valuable research directions as well as current challenges. The annual publication trends unveil the different developmental stages of AI and head and neck cancer. Before 2006, research was still scattered, as AI technology was in its infancy, computational power and data resources were limited, and the medical field was taking a wait-and-see attitude toward its application, with research largely confined to theoretical discussions. In 2017 and 2018, researchers began to focus on the potential of AI in medical imaging analysis of head and neck cancer, marking the initial entry of AI technology into the stage of medical practice verification. After 2019, the number of publications rapidly increased, and academic recognition significantly improved. Relevant research will continue to grow rapidly and gradually deepen into clinical practice.

We conducted a quantitative analysis of three structural levels: countries, institutions, and authors, and generated a co-occurrence map of collaborations. The United States has a long history of conducting research on AI in head and neck cancer, with a significant number of high-quality publications. This research is primarily concentrated in large public university systems and top medical schools, such as the University of Texas System and the University of California System. These institutions frequently collaborate with European organizations, emphasizing cross-disciplinary cooperation and forming a global collaboration network led by the US. Additionally, the US holds a technological leadership position in the field of AI and head and neck cancer due to its early technological advantages, including original underlying algorithms and substantial government funding (21). In recent years, China has experienced rapid development in this field, with a sharp increase in the number of publications. China has a high incidence of head and neck cancer globally (2), and the significant clinical demand has driven the development of localized AI tools. The abundance of clinical data provided by the public hospital system, resulting from the large number of patients, is a crucial factor in China’s rapid progress in AI and head and neck cancer research. However, Chinese institutions tend to favor independent research or domestic collaborations, with inward-looking cooperation as the dominant approach. Therefore, their international influence still needs to be enhanced. We believe that in the future, the US and China will follow differentiated paths in AI for head and neck cancer research, with the US focusing on “technological leadership” and China on “scale catch-up,” jointly promoting the field’s development through competition and cooperation in data sharing, algorithm validation, clinical trials, and standard-setting, establishing a two-way mechanism from technical design to clinical validation. Chinese datasets and clinical validation pipelines could enhance the generalizability of AI models developed in the West, while U.S.-led innovations can benefit from testing at scale in China’s extensive clinical networks. A more synergistic relationship—balancing competition and cooperation, algorithmic excellence with large-scale clinical validation—has the potential to accelerate global progress in AI-assisted head and neck cancer care. Peretti, Giorgio, and Sampieri, Claudio, have the highest number of publications and are leading scholars in the application of AI to head and neck cancer. They are also otolaryngologists at the University of Genoa. They frequently collaborate with the Department of Advanced Robotics at the Istituto Italiano di Tecnologia and the Department of Otolaryngology at Hospital Clínic in Barcelona, leveraging the strengths of both engineering and medical teams to advance the implementation of AI technologies in head and neck cancer research. Their work primarily focuses on innovative applications of AI in endoscopy and laryngoscopy, achieving automatic tumor segmentation in endoscopic images and significantly improving the accuracy of boundary identification in throat and oropharyngeal cancers (22). They have also explored the use of AI in clinical decision-making, comparing the consistency of treatment recommendations between ChatGPT and the NCCN guidelines for head and neck cancer, and investigating the potential of large language models (LLMs) in personalized therapy (23).

*Radiotherapy And Oncology* and *Cancers* are the most frequently cited journals, indicating their significant academic influence. *Radiotherapy And Oncology* focuses on cutting-edge research integrating radiotherapy and oncology, with a particular emphasis on the transformative applications of AI in radiotherapy planning optimization, organ segmentation, adaptive radiotherapy, and prognosis prediction. In 2019, Kosmin, M. published a review in this journal discussing the use of AI for automatic segmentation techniques in head and neck cancer radiotherapy, which has been cited over 100 times, cementing AI’s central role in radiotherapy planning (24). On the other hand, *Cancers* spans the entire field of cancer, emphasizing multidisciplinary approaches. Its AI research centers on exploring fundamental mechanisms and clinical translation, such as medical and pathological image analysis (25), early screening for oral cancer (26), and HPV-related oropharyngeal cancer prediction (27).

This study used keyword clustering analysis and burst analysis. In recent years, a growing number of scholars have conducted comprehensive and systematic research on the application of AI in the field of head and neck cancer, covering various aspects such as medical imaging, radiation therapy, automatic diagnosis systems, and predictive models. The emergence of recent burst words like “target volumes”, “prognosis”, “algorithm”, “survival”, “lesions”, and “automatic diagnosis”, signifies three paradigm shifts in the diagnosis and treatment of head and neck cancer using AI: 1. Transition from experience-driven to data-driven precision therapy: AI replaces traditional target volume delineation to achieve precise radiotherapy, integrating clinical data to construct multifactorial predictive models for personalized treatment plans (28). 2. Evolution from manual operation to full-process automation: AI enables automatic detection of lesions, gradually realizing automatic diagnosis (29). Recent advances have shown that AI can assist in the early screening of head and neck cancer, particularly in primary care settings. By integrating image analysis (tissue sections, photographic images) with the clinical demographic information of patients (such as age, smoking and drinking history, lesion size, location), the classification accuracy of AI models for oral leukoplakia and OSCC has significantly improved (combined data accuracy ≈ 95%, only with images ≈ 93%; when distinguishing whether there is mucosal dysplasia, the accuracy rate has increased to 88% vs. 83%) (30). This application highlights the potential of artificial intelligence as a frontline screening aid, which can enhance early identification and improve access to timely specialist care, benefiting general practitioners in screening and subsequent specialist treatment. 3. Shift from static analysis to dynamic survival management: While traditional methods predict patient survival rates based on TNM staging, AI introduces real-time dynamic indicators (such as tumor size, EBV DNA, and albumin) to enable dynamic risk stratification (31)

Although AI has revolutionized the diagnosis and treatment of head and neck cancers, its development still faces significant technical, clinical, and regulatory challenges that must be addressed to promote its responsible use in real-world clinical settings.

Firstly, the training, validation, and testing of AI models rely on learning from large-scale, high-quality, and diverse databases. The size, quality, and accessibility of these databases have a direct impact on the performance of AI models (32). Therefore, it is crucial to establish standardized protocols for data collection, annotation, and reporting, as well as promote the integration and sharing of multi-center databases across institutions and countries. Enhancing AI models’ multimodal learning from different patient populations and various omics-derived data is essential to improve their generalization ability, address potential algorithmic biases, and enhance their accuracy and robustness (30, 33). In addition to data standardization, the standardization of AI models themselves is also vital. Research and development institutions should strive to coordinate the standardization of AI models during their development and deployment. Benchmarking algorithms and developing unified guidelines for model performance metrics and application standards are necessary to ensure that AI models undergo comprehensive fairness and bias testing before implementation, guaranteeing their fairness, safety, and reliability (30, 34).

Secondly, the structure and operational principles of deep learning models are often difficult to explain, earning them the moniker of “black boxes” (35).Therefore, the development of explainable AI is crucial for providing a clear and transparent decision-making process that helps clinicians understand and trust AI decisions. This not only fosters effective communication between clinicians and AI but also aids in identifying potential biases in AI models, ensuring the safety and effectiveness of AI decisions (36). By forming a closed-loop verification process through visualization of model architectures, mapping of medical features, and clinician feedback, explainable AI can gradually break down the barriers of the “black box” and become a trusted intelligent assistant in the diagnosis and treatment of head and neck cancers.

Finally, the application of AI in the field of head and neck cancer requires regulation by ethics, morals, and laws. As the training process of AI models involves the use of patients’ clinical data, medical regulatory bodies should vigorously promote the establishment of relevant laws, regulations, and ethical guidelines. They should also develop robust data encryption methods and sharing protocols to ensure the security and confidentiality of data during its use and sharing (37). In the event of medical accidents arising from AI-guided diagnosis and treatment, the attribution of responsibility becomes a complex legal, ethical, and technical issue. The challenges in attributing liability for AI-related medical incidents include: difficulty in proving causation due to multiple contributing factors, the “black box” nature of AI complicating accountability tracing, and transnational legal disparities in AI governance. However, core principles for AI liability allocation remain clear: developers bear responsibility for technical flaws, physicians for clinical decisions, and medical institutions for system management. Future efforts should focus on legal refinement, technological transparency, and ethical standardization to establish a collaborative accountability framework that balances innovation with patient safety. In the future, it is necessary to build a multi-party collaborative responsibility framework through legal improvement, technical transparency, and ethical norms, to balance innovation and patient safety. Algorithm bias refers to the systematic unfairness of AI systems toward specific groups during data processing, model training, or decision-making, due to limitations in data, design, or application scenarios (38). This bias is not “intentional discrimination” but rather an accumulation of implicit defects in multiple links of the technical chain (38).The author speculates that in the field of head and neck cancer, algorithmic bias may manifest as tendency biases in treatment recommendations, diagnostic accuracy disparities across demographic groups, and unequal deployment of AI-driven early screening between affluent and underserved regions. Algorithm bias is not only a technical issue but also a reflection of social structural inequalities. If left unchecked, AI could transform from being a “universally beneficial tool” into a “destroyer of medical equity. The research trends identified in this study provide guidance for reducing bias in AI development and deployment within head and neck cancer contexts. For instance, analyzing demographic bias patterns in hotspot areas like “survival prediction” and “lesion identification” can inform the development of fairer algorithms. In short, as artificial intelligence transitions from decision support to clinical applications in head and neck cancer, its three core ethical and legal challenges are: 1) Balancing data utility and patient privacy protection during model training; 2) Clarifying the chain of responsibility in misdiagnosis incidents, especially when the opacity of deep learning models conflicts with the final decision-making power of clinical doctors; 3) Eliminating diagnostic biases caused by insufficient representativeness of training data through algorithm auditing. Solving these problems systematically will promote the sustainable development of artificial intelligence in tumor diagnosis and treatment practices.

However, even if these core issues are resolved, AI still faces four key obstacles from research to clinical practice: Firstly, there is uncertainty in how AI models will perform when extended to different patient populations (33), especially insufficient validation for patients with ethnic minorities or rare diseases, and it is urgent to establish inclusive clinical trial standards. For instance, AI-assisted diagnostic and therapeutic modeling for rare subtypes (39) currently faces challenges such as limited sample sizes and high heterogeneity, which conventional AI models struggle to address effectively. Secondly, the regulatory system has insufficient adaptability to continuous learning algorithms, and an iterative certification process needs to be designed. Thirdly, primary medical institutions lack computing infrastructure such as GPU servers and standardized imaging equipment. Fourth, clinicians’ attitudes toward AI are polarized, and the cognitive gap needs to be bridged through human-machine collaboration training.

At present, the long-term prognosis of head and neck cancer patients receiving artificial intelligence-assisted treatment remains unclear. To transform artificial intelligence from theoretical models into clinically valuable applications, large-scale, multi-center, and prospective studies are needed in the future to verify its stability, compatibility, and reproducibility in real clinical settings (40). Additionally, the application of artificial intelligence in predicting treatment-related adverse reactions and guiding postoperative rehabilitation still requires in-depth exploration. Future research should break through the limitation of merely optimizing treatment plans and expand into the field of complication prediction modeling. By integrating multi-modal data such as imaging, genomics, epigenetics, and immunomics, and promoting interdisciplinary collaboration in multi-center model training, while enhancing cross-language and cross-cultural universality, artificial intelligence platforms will achieve revolutionary improvements in usability and interpretability.

In conclusion, the integration of artificial intelligence into comprehensive management of head and neck cancer holds the potential to revolutionize the diagnostic and therapeutic landscape in this field. While current challenges persist regarding standardization of medical datasets and AI models, interpretability of algorithmic decision-making, validation through clinical efficacy studies, and unresolved ethical-legal considerations, accumulating evidence suggests that continued refinement of AI technologies will progressively enhance their clinical utility. This technological evolution promises to deliver tangible benefits for both patients and clinicians through optimized decision support systems and precision medicine applications in head and neck oncology.

## 5 Limitation

1 Inherent limitations of bibliometric methodology and database selection: Bibliometric analyses are inherently constrained by the scope and biases of the chosen database. While WoSCC was selected for its comprehensive coverage of high-quality medical literature, it excludes certain types of publications (e.g., preprints, conference abstracts, gray literature, and non-indexed journals) that may contain emerging research relevant to AI in head and neck cancer. This selectivity potentially limits the representativeness of the dataset, as studies from under-resourced regions or early-stage investigations—often published in non-indexed outlets—may be underrepresented. Additionally, WoSCC’s indexing criteria (e.g., focus on English-language, peer-reviewed journals) inherently skews the dataset toward research from institutions with greater publication resources, which may affect the generalizability of conclusions regarding global research trends.

2 Language and keyword-related biases: Our restriction to English-language articles introduces a language bias, as non-English research (e.g., regional studies in high-incidence areas like Southeast Asia) is excluded. While such articles constitute <2% of WoSCC records and often lack standardized formatting, their omission may obscure localized innovations. Furthermore, keyword-based retrieval is inherently dependent on the specificity of search terms (“artificial intelligence,” “head and neck cancer,” etc.). This approach may miss studies using synonymous terminology (e.g., “machine learning” vs. “AI,” “oropharyngeal malignancy” vs. “head and neck cancer”) or interdisciplinary jargon, potentially excluding relevant literature and limiting the comprehensiveness of the dataset.

3 Retrieval and screening biases: Reliance on topic-based searches (rather than full-text screening) and manual exclusion of irrelevant articles introduces subjectivity, despite the triple-blind evaluation process. While efforts were made to standardize inclusion/exclusion criteria, individual interpretable differences in judging “relevance” to AI or head and neck cancer may have introduced selection bias. Additionally, the focus on original articles excludes reviews, editorials, and case reports, which—though not primary research—often synthesize key trends or highlight clinical challenges, potentially narrowing the analytical scope.

4 Analytical focus limitations: This study focuses on mapping macro-level trends (hotspots, collaboration networks, keyword clusters) rather than evaluating the methodological rigor or clinical validity of individual studies. As such, it cannot address whether high-frequency research areas (e.g., AI-driven radiotherapy) align with actual clinical needs or whether reported algorithms are reproducible in real-world settings. This limitation underscores that bibliometric insights into “research intensity” do not equate to evidence of “clinical impact.

## Data Availability

The original contributions presented in the study are included in the article/**Supplementary Material**. Further inquiries can be directed to the corresponding author.
