# Supplementary material for: Artificial intelligence in head and neck cancer: a bibliometric analysis of research landscape, emerging trends, and challenges
Source: Front Oncol. 2025 Sep 1;15:1604136. doi: 10.3389/fonc.2025.1604136 (PMC12434015; doi:10.3389/fonc.2025.1604136)
Supplement: Supplementary file 1 [file DataSheet1.pdf]

Supplementary material

Our bibliometric analysis reveals several notable gaps between research focus and clinical implementation, highlighting areas where academic progress has outpaced real-world adoption. These discrepancies underscore critical barriers that must be addressed to bridge the "translational gap" in AI-driven head and neck cancer management.AI models for automated tumor segmentation, dose prediction, and imaging-based diagnosis dominate the literature, with robust algorithmic advancements reported. However, clinical implementation lags for the following reasons.First, there is technical fragmentation. Most models are trained on single-center datasets, lacking generalizability across diverse patient populations or clinical settings (e.g., varying imaging protocols between institutions). AI tools often prioritize algorithmic performance over clinical usability (e.g., integration with existing hospital information systems), which may create friction in busy oncology workflows.(1-4) Additionally, despite promising technical outcomes, few AI imaging/radiotherapy tools have obtained rigorous regulatory approval (e.g., FDA clearance), limiting widespread adoption.From keyword distributions and national/institutional collaboration networks, we observe limited research focus on AI applications for early detection of occult head and neck lesions (e.g., in asymptomatic populations) or deployment in resource-constrained settings—despite these being critical unmet clinical needs (particularly in high-incidence regions like Southeast Asia, which are underrepresented in publication maps).Potential reasons, in our view, include the underrepresentation of early-stage lesions in existing datasets, which hinders model development. Moreover, technology developers tend to prioritize their own high-resource contexts (e.g., tertiary hospitals with advanced imaging capabilities) during development, while overlooking tools adapted for low-cost modalities (e.g., mobile endoscopy) or settings with limited connectivity.

References

1. Werth K, Ledbetter L. Artificial Intelligence in Head and Neck Imaging a Glimpse into the Future. *Neuroimaging Clinics of North America* (2020) 30(3):359-+. doi: 10.1016/j.nic.2020.04.004.
2. Rao KN, Fernandez-Alvarez V, Guntinas-Lichius O, Sreeram MP, de Bree R, Kowalski LP, et al. The Limitations of Artificial Intelligence in Head and Neck Oncology. *Advances in Therapy* (2025) 42(6):2559-68. doi: 10.1007/s12325-025-03198-4.
3. Pham TD, Teh M-T, Chatzopoulou D, Holmes S, Coulthard P. Artificial Intelligence in Head and Neck Cancer: Innovations, Applications, and Future Directions. *Current Oncology* (2024) 31(9):5255-90. doi: 10.3390/curroncol31090389.
4. Alabi RO, Almangush A, Elmusrati M, Leivo I, Makitie AA. Implementation Challenges of Artificial Intelligence-Based Radiomics in Head and Neck Oncology: A Systematic Review. *Radiotherapy and Oncology* (2024) 192:S207-S9.
